# Supplementary material for: Motivated for near impossibility: How task type and reward modulate task enjoyment and the striatal activation for extremely difficult task
Source: Cogn Affect Behav Neurosci. 2022 Nov 30;23(1):30–41. doi: 10.3758/s13415-022-01046-4 (PMC9925569; doi:10.3758/s13415-022-01046-4)
Supplement: Supplementary file 1 — (DOCX 410 kb) [file 13415_2022_1046_MOESM1_ESM.docx]

*Table S1.* Results of 3 x 3 whole-brain ANOVA

| Cluster | Cluster size (number of voxels) | AAL region | MNI peak coordinates | | | peak F statistic |  |
| --- | --- | --- | --- | --- | --- | --- | --- |
|  |  |  | x | y | z |  |  |
| **Main Effect of Group** | | | | | | | |
| No significant activations | | |  |  |  |  |  |
| **Main Effect of Chance of Success** | | | | | | | |
| 1 | 71 | R Temporal Lobe | 34 | -50 | 4 | 20.73 |  |
| 2 | 112 | L Cerebellum | -12 | -46 | -48 | 18.97 |  |
| 3 | 33 | L Calcarine Gyrus | -28 | -72 | 8 | 18.48 |  |
| 4 | 25 | R Cuneus | 18 | -84 | 32 | 16.06 |  |
| 5 | 8 | R. Cerebellum | 28 | -46 | -42 | 15.89 |  |
| **Interaction Effect Group x Chance of Success** | | | | | | | |
| 1 | 44 | Right VS/VP | 10 | 4 | -10 | 11.82 |  |
| 2 | 46 | R Lingual Gyrus | 16 | -90 | -14 | 10.93 |  |
| 3 | 32 | L Calcarine Gyrus | -2 | -90 | -10 | 10.93 |  |
| 4 | 53 | R Cerebellum | 20 | -84 | -28 | 10.09 |  |
| 5 | 9 | L Lingual Gyrus | -16 | -92 | -18 | 9.90 |  |
| 6 | 6 | Left VS/VP | -8 | 6 | -8 | 9.56 |  |
| *Note*. All *ps* < .05, cluster-level FWE corrected (with the initial thresholding at p < .001). | | | | | | | |

*Table S2.* Results of gPPI analysis with the ventral striatum/ventral pallidum seed, comparing reward and no-reward groups for extremely-low chance of success.

| Cluster | Cluster size (number of voxels) | AAL region | MNI peak coordinates | | | peak t statistic |  |
| --- | --- | --- | --- | --- | --- | --- | --- |
|  |  |  | x | y | z |  |  |
| **Seed: Right VS/VP, reward group > no-reward group** | | | | | | | |
| No significant activations | | |  |  |  |  |  |
| **Seed: Right VS/VP, no-reward group > reward group** | | | | | | | |
| No significant activations | | |  |  |  |  |  |
| **Seed: Left VS/VP, reward group > no-reward group** | | | | | | | |
| 1 | 267 | L Putamen | -22 | 12 | 8 | 6.04 |  |
| 2 | 564 | R Cerebellum | 30 | -64 | -54 | 5.47 |  |
| 3 | 588 | L Middle Frontal Gyrus | -36 | 30 | 40 | 5.41 |  |
|  |  | L Somatosensory Area | -28 | 14 | 32 |  |  |
| 4 | 828 | L Inferior Frontal Gyrus | -48 | 40 | -6 | 5.35 |  |
|  |  | L Middle Orbital Gyrus | -32 | 42 | -8 |  |  |
| **Seed: Left VS/VP, no-reward group > reward group** | | | | | | | |
| No significant activations | | | | | | | |
| *Note*. All *ps* < .05, cluster-level FWE corrected (with the initial thresholding at *p* < .0025). | | | | | | | |


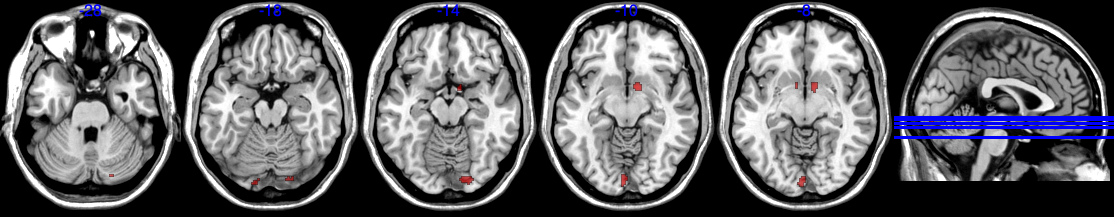


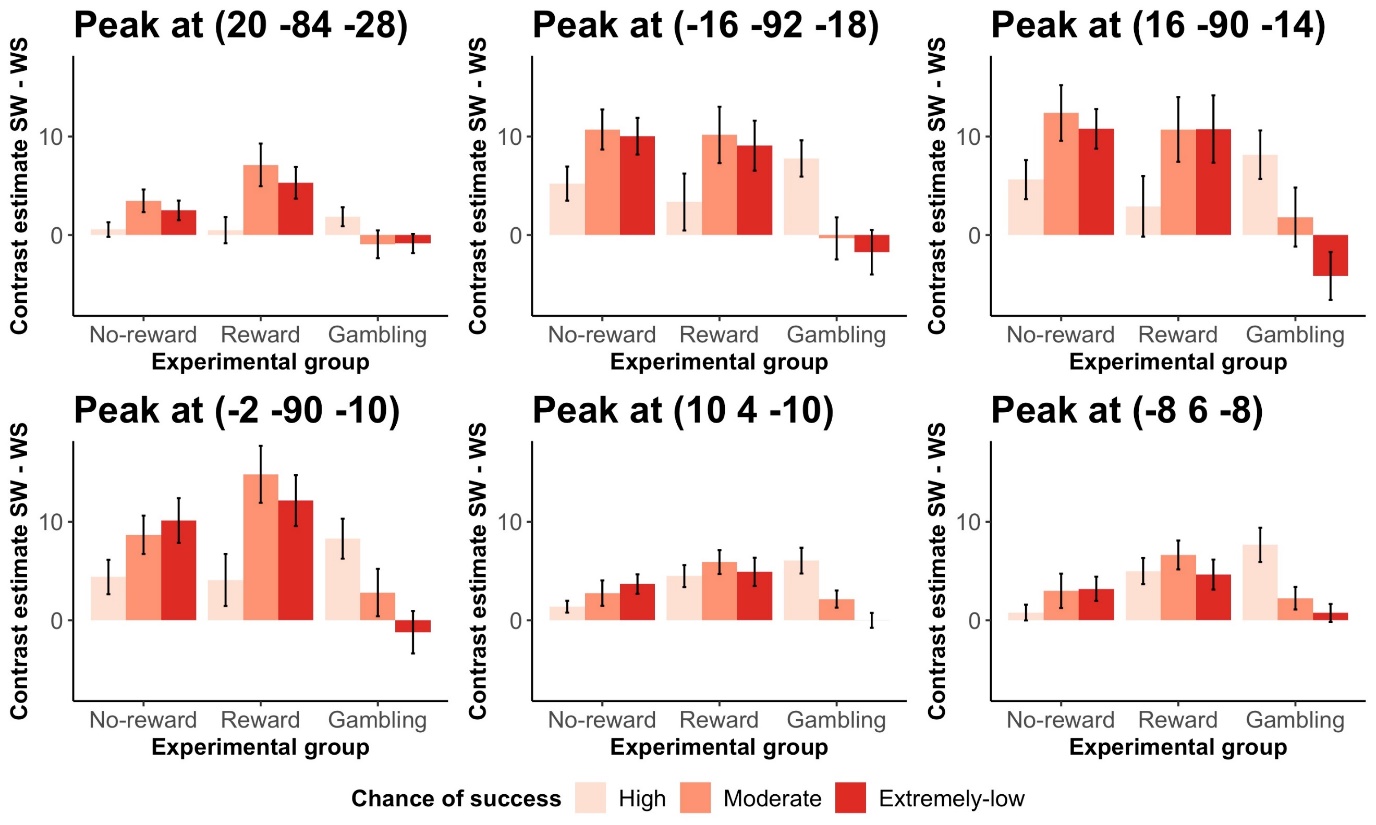


**Figure S1** Activation pattern across conditions and groups for the peak voxels of the clusters that showed a significant 2-way interaction (with activation map to indicate the exact locations).
